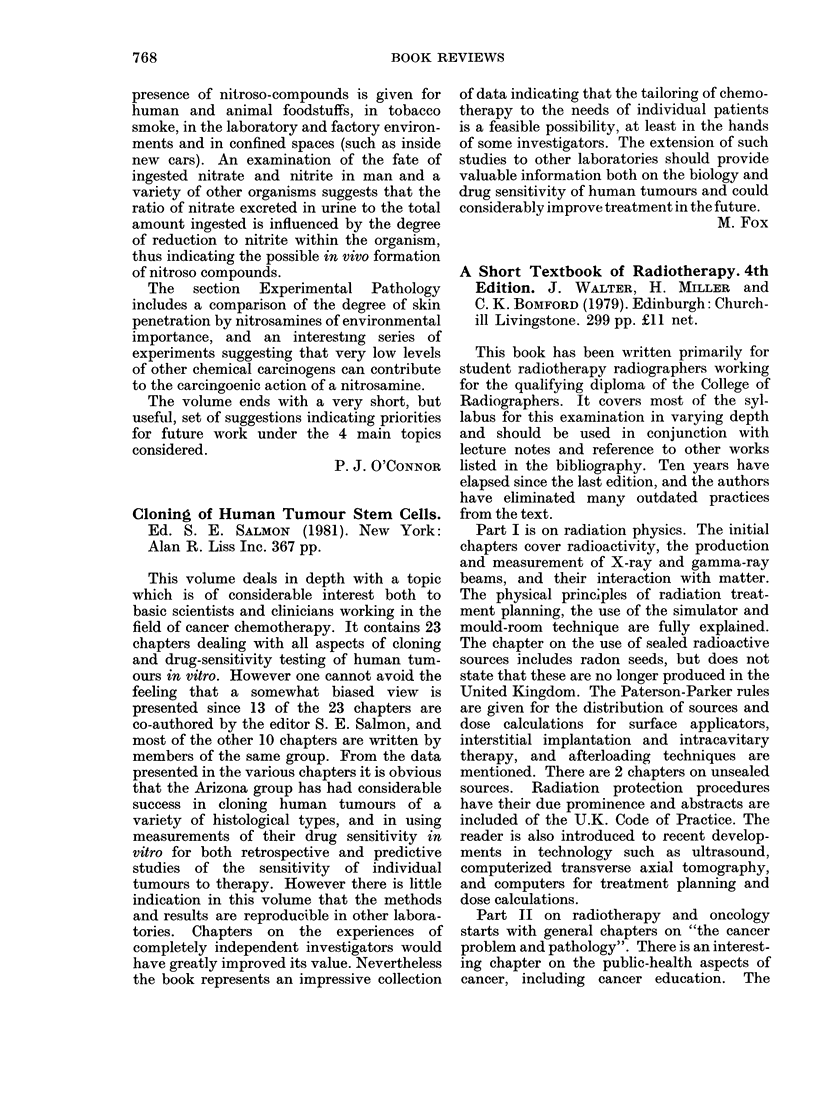# Cloning of Human Tumour Stem Cells

**Published:** 1981-11

**Authors:** M. Fox


**Cloning of Human Tumour Stem Cells.** Ed. S. E. SALMON (1981). New York: Alan R. Liss Inc. 367 pp.

This volume deals in depth with a topic which is of considerable interest both to basic scientists and clinicians working in the field of cancer chemotherapy. It contains 23 chapters dealing with all aspects of cloning and drug-sensitivity testing of human tumours *in vitro*. However one cannot avoid the feeling that a somewhat biased view is presented since 13 of the 23 chapters are co-authored by the editor S. E. Salmon, and most of the other 10 chapters are written by members of the same group. From the data presented in the various chapters it is obvious that the Arizona group has had considerable success in cloning human tumours of a variety of histological types, and in using measurements of their drug sensitivity *in vitro* for both retrospective and predictive studies of the sensitivity of individual tumours to therapy. However there is little indication in this volume that the methods and results are reproducible in other laboratories. Chapters on the experiences of completely independent investigators would have greatly improved its value. Nevertheless the book represents an impressive collection of data indicating that the tailoring of chemotherapy to the needs of individual patients is a feasible possibility, at least in the hands of some investigators. The extension of such studies to other laboratories should provide valuable information both on the biology and drug sensitivity of human tumours and could considerably improve treatment in the future.

M. FOX